# NLOS Multipath Classification of GNSS Signal Correlation Output Using Machine Learning

**DOI:** 10.3390/s21072503

**Published:** 2021-04-03

**Authors:** Taro Suzuki, Yoshiharu Amano

**Affiliations:** 1 Future Robotics Technology Center, Chiba Institute of Technology, Chiba 2750016, Japan; 2 Department of Applied Mechanics and Aerospace Engineering, Waseda University, Tokyo 1620044, Japan; yoshiha@waseda.jp

**Keywords:** GPS, GNSS, SDR, multipath, signal classification, machine learning

## Abstract

This paper proposes a method for detecting non-line-of-sight (NLOS) multipath, which causes large positioning errors in a global navigation satellite system (GNSS). We use GNSS signal correlation output, which is the most primitive GNSS signal processing output, to detect NLOS multipath based on machine learning. The shape of the multi-correlator outputs is distorted due to the NLOS multipath. The features of the shape of the multi-correlator are used to discriminate the NLOS multipath. We implement two supervised learning methods, a support vector machine (SVM) and a neural network (NN), and compare their performance. In addition, we also propose an automated method of collecting training data for LOS and NLOS signals of machine learning. The evaluation of the proposed NLOS detection method in an urban environment confirmed that NN was better than SVM, and 97.7% of NLOS signals were correctly discriminated.

## 1. Introduction

**Figure 1:**
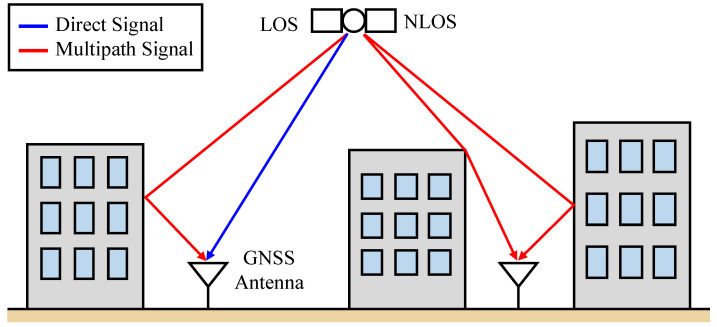
Comparison of line-of-sight (LOS) and non-LOS (NLOS) multipath signals. If a global navigation satellite system (GNSS) receiver receives reflected or diffracted signals from an “invisible” satellite behind a building, a large positioning error will occur.

The global navigation satellite system (GNSS) is currently used in various location-based services. As of 2020, various countries have launched and are operating their own positioning satellites, and there are approximate 30 satellites available for positioning on average, and thus the availability of satellite positioning is expected to significantly increase. One of the most sought-after location-based services is stable positioning in urban environments with many buildings. However, despite the increase in the number of positioning satellites, large positioning errors occur suddenly in urban environments. This positioning error is caused by multipath signals, where GNSS signals are reflected or diffracted by buildings and other objects [1]. There are two types of multipath, between which the effect on the GNSS receiver is significantly different. Figure 1 illustrates the line-of-sight (LOS) and non-line-of-sight (NLOS) multipath signals. GNSS antennas receive direct signals and reflected or diffracted signals simultaneously, which is called LOS multipath. The reflected or diffracted signals affect the correlation process of the direct signal, arriving at the antenna first and deteriorate the ranging accuracy of GNSS. This ranging error is called LOS multipath error. However, this error can be mitigated by devising a signal correlation processing [2,3,4]. Owing to an innovation of the signal correlation method, the LOS multipath error within the GNSS pseudorange is only a few meters at most, and the maximum error has an upper limit.

On the other hand, in an urban environment with many buildings, GNSS signals emitted from out-of-sight satellites hidden behind buildings are often received by the antennas through reflection and diffraction. In this case, a larger-than-normal error occurs in GNSS positioning [5]. This is called an NLOS multipath error; in addition, the magnitude of the error depends on the environment, and the maximum error cannot be defined.

Because the pseudorange observed by the NLOS signal is heavily biased against the true distance, the positioning results are usually significantly deteriorated when the pseudorange of the NLOS signal is used for positioning calculations [6]. This pseudorange bias is difficult to compensate, and thus, detecting the NLOS signal and excluding it from the positioning calculation is the easiest way to improve the positioning accuracy.

A method of NLOS discrimination using GNSS receiver output is proposed for NLOS discrimination using signal-to-noise ratio (SNR) [7,8,9]. This method takes advantage of the phenomenon that reflected and diffracted NLOS signals have a lower signal strength compared to direct signals. However, it is difficult to correctly discriminate between NLOS signals when a diffractive signal near the edge of a building or a reflected signal such as a specular reflection is received.

In this study, we proposed a novel NLOS multipath detection method that can employ machine learning techniques to enhance the GNSS positioning performance in urban environments, where NLOS multipath signals lead to major positioning errors. The basic idea of the proposed method is to integrate a discriminator in the GNSS signal processing that discriminates NLOS from the results of the GNSS signal correlation outputs instead of discriminating NLOS from GNSS observations such as SNR. The GNSS signal correlation result is the primary GNSS output and contains information on NLOS signals. The proposed method learns the “features of the shape of the correlation function”, which is the output of GNSS signal correlation processing. We propose two supervised learning methods, one with a support vector machine (SVM) and the other with a neural network (NN), and compare their levels of performance.

### 1.1. Related Studies

NLOS multipath errors have long been studied as a serious problem in GNSS positioning. The countermeasures against NLOS multipath errors can be classified into four categories: (1) the rejection of GNSS positioning results that contain such errors, (2) the identification of NLOS signals from GNSS observations and their exclusion from the positioning calculations, (3) the estimation and correction of NLOS multipath errors, and (4) the suppression of NLOS reception in the RF section. The latter method is a more fundamental countermeasure for NLOS multipath errors.

(1) The rejection of GNSS positioning results containing NLOS multipath errors is a countermeasure against such errors. Several methods have been proposed to detect jumps in GNSS positioning, such as methods using a Chi-square test [10] in positioning calculations, and methods that combine the trajectories estimated by other sensors such as IMU, wheel odometry, and visual odometry using cameras [6]. However, these methods are impractical and fundamental measures because they reduce the availability of GNSS positioning.

(2) NLOS signals are identified from GNSS observations and excluded from the positioning calculation, which is the most studied NLOS multipath reduction method. Receiver autonomous integrity monitoring (RAIM)-related techniques are used to detect large GNSS ranging errors in the observed GNSS pseudorange [11]. However, these methods assume that the number of NLOS signals is extremely small among the received signals, and thus they cannot be used in an environment in which there are many NLOS signals. Methods for detecting an NLOS from GNSS observations using a visible camera [12], a fish-eye camera [13], and an omnidirectional far-infrared camera [14] have been proposed. The camera is pointed toward the zenith to detect an NLOS satellite hidden in a building. However, NLOS detection using a camera is affected by weather and illumination conditions. An omnidirectional far-infrared camera is more robust than previously proposed methods. However, it requires special sensors, and a camera-based method is required to measure the camera pose to project the satellite position onto the image. Real-time laser scanning is also used to detect NLOS signals [15,16]. However, there is a limitation to the measurement range of the laser scanner. 3D maps have attracted attention in improving the GNSS positioning accuracy in urban environments [17,18,19,20]. A 3D city model can be used to detect NLOS signals combined with GNSS receivers. However, an accurate 3D model is needed to compute the position in advance.

(3) An estimation and correction is an ideal countermeasure to deal with NLOS multipath errors. If an NLOS signal is rejected in an urban environment, problems such as an insufficient number of satellites and a deterioration of the satellite geometry will occur. A few methods for correcting NLOS multipath errors using 3D maps have been proposed [21,22]. However, the correction of NLOS multipath errors is extremely complex and difficult and is not yet practical in terms of accuracy.

(4) Suppression of the NLOS reception in the RF section is a more fundamental countermeasure for NLOS multipath errors. For example, the use of a choke ring antenna [23] and a dual-polarization antenna [24,25] has been proposed. The reflected signal is changed from a right-hand circularly polarized signal to a left-hand circularly polarized signal. Using the dual-polarization antenna, we can separately process both signals, and the reflected signal can be estimated from the difference of the SNRs of the right- and left-hand signals. The use of this method comes with associated problems such as the system complexity and operational costs.

By contrast, recent developments in machine learning techniques have led to significant research into improving the GNSS positioning accuracy through machine learning [26,27,28,29,30,31]. In [26,28,30], a decision tree or SVM is used for discriminating NLOS signals from learning of GNSS observations, such as SNRs and pseudoranges. In [29], the indoor LOS multipath signal classification based on a deep learning approach was proposed. However, this work focuses on the LOS multipath detection of a pseudolite and is difficult to apply to NLOS detection in outdoor environments. In [31], a convolutional neural network (CNN)-based NLOS detection approach was proposed. This study also uses GNSS observations for NLOS detection and did not use the signal correlation output. In our previous study [27], we formed the basis of the present study by incorporating machine learning methods into a GNSS software receiver and proposing a method for discriminating NLOS signals from the correlation outputs based on an SVM. Compared to the previous study, the present approach utilizes multiple machine learning methods, SVM and NN, the discrimination performances of which are compared herein.

### 1.2. Contributions

The contributions of this paper are as follows.

- The idea of direct machine learning of a GNSS signal correlation output, which is the most primitive GNSS signal processing output, is an innovative approach.
- In this study, multiple machine learning methods (e.g., SVM and NN) are applied to compare the performance of an NLOS signal detection. There have been few examples of applying an NN to GNSS signal processing, and NLOS discrimination using an NN has been a significant achievement.
- We devised a data acquisition device that automatically collects labeled NLOS signals to collect training data, which is a problem in supervised machine learning.
- We applied the proposed machine learning method to GNSS signals acquired in a real urban environment.

The remainder of this paper is organized as follows: First, in Section 2, we describe the details of the proposed method and the basic principles of the proposed method for detecting the NLOS. Then we describe a method for detecting the NLOS using an SVM and NN. In Section 3, we describe a data acquisition device that automatically collects training data for machine learning. In Section 4, we describe an evaluation of NLOS multipath detection in the actual environment. Finally, we present a discussion and conclusion.

## 2. Proposed Method

### 2.1. Outline of Proposed System

**Figure 2:**
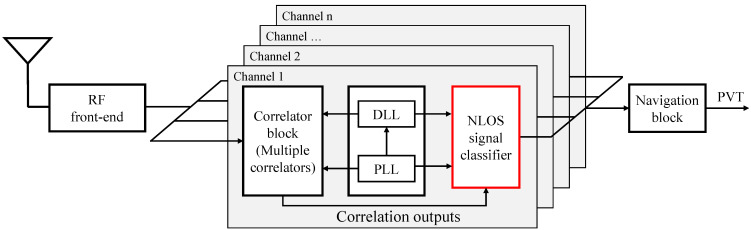
Outline of the proposed system. We incorporated an NLOS discriminator into the conventional GNSS signal processing algorithm, which directly discriminates the NLOS signal from the correlation output and functions as a filter that outputs only the LOS signal.

In this study, an NLOS multipath discriminator using a GNSS signal correlation output is implemented in a software GNSS receiver. Figure 2 shows the outline of the proposed system. The GNSS signal input from the antenna is digitized in the RF front end and sent to the baseband processing. In the baseband processing, the input digitized signal is acquired and tracked by calculating the correlation with the replica code generated in the receiver on an independent channel for each satellite. In the correlation process within GNSS receivers, the correlation of incoming signals and the replica code generated in the GNSS receiver is computed. This is called GNSS signal correlators. We used multiple correlators to extract NLOS correlation features instead of the standard early late-prompt correlator. In the delay lock loop (DLL), the code correlation peak is determined and tracked using the discriminator computed by the outputs of the signal correlators. The GNSS carrier phase is also tracked in the phase-locked loop (PLL). Then, GNSS observations such as the pseudoranges, carrier phase, and Doppler frequency are computed from the result of the code tracking in each channel [1]. Finally, the position, velocity, and time (PVT) are computed from GNSS observations. Here, an NLOS signal classifier created based on a machine learning technique is added after the loop filter block. The input of the NLOS classifier is the output of the signal correlators. The NLOS classifier determines whether the signal currently being tracked by the channel is LOS or NLOS from the input correlation output. If it is an NLOS, it acts as a filter that rejects GNSS observations instead of outputting them. Hence, only LOS signals are used for positioning in the navigation block, and thus the positioning accuracy in an urban environment can be improved. In addition, the proposed method does not require additional sensors and is practical and easy to implement.

Various classification methods using machine learning have been proposed. In this study, supervised machine learning methods, SVM and NN, are applied to the NLOS classification problem. An SVM uses a kernel trick technique to transform the input data to find the optimal bounds of the target classes. The designers of a discriminator need to extract the NLOS features from the input signal themselves, and the SVM classifies the signal based on the extracted features. For this reason, it is important for an SVM to determine features that can fully explain the NLOS signal correctly. By contrast, an NN is frequently used in classification problems. An NN is a computational model inspired by the manner in which biological neural networks in the human brain process information. Unlike an SVM, in an NN, features for classification are automatically acquired during the learning process. Therefore, the performance does not depend on the features extracted by the designer, as in an SVM. However, an NN generally requires a large number of training data. The method for obtaining a labeled NLOS signal for training is described in Section 5. In this study, we use the features of an NLOS, as described in the next section, to determine the feature extraction of an SVM and the input of an NN and compare the performance of each supervised machine learning method.

### 2.2. NLOS Correlation Function

A feature of the proposed method is that it discriminates the NLOS signal by directly learning the correlation output of the NLOS multipath signal; the correlation function of the NLOS multipath signal is significantly distorted compared to an LOS multipath signal with a direct signal. First, the shape of the correlation function for the LOS and NLOS multipath signals is clarified. We denote C(t) the GNSS pseudo-random noise (PRN) code sequence. The amplitude, carrier frequency, and signal delay of a direct signal are denoted as $A_{0}$, $\omega_{0}$, $\tau_{0}$, and $\theta_{0}$, respectively. A direct GNSS signal $S_{0}\left(t\right)$ can be denoted as follows:

(1) $$S_{0}\left(t\right)=A_{0}\cdot C\left(t-\tau_{0}\right)\cdot \cos\left(\omega_{0}t\right)$$

**Figure 3:**
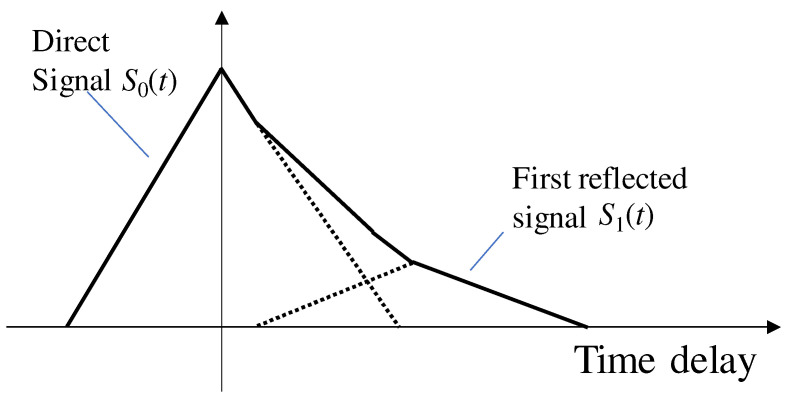
Correlation function of an LOS multipath signal.

The case in which the direct signal $S_{0}\left(t\right)$ is affected by the single reflected signal $S_{1}\left(t\right)$. The LOS multipath signal $S_{\mathrm{LOS}}\left(t\right)$ can be expressed as follows:

(2) $$\begin{array}{cc} S_{\mathrm{LOS}}\left(t\right) & =S_{0}\left(t\right)+S_{1}\left(t\right)\\ \; & =S_{0}\left(t\right)+A_{1}\cdot C\left(t-\tau_{1}\right)\cdot \cos\left(\omega_{0}t+\Delta \phi_{1}\right). \end{array}$$

where $A_{1}$, $\tau_{1}$, and $\Delta \phi_{1}$ are the multipath amplitude, delay, and relative phase between direct and multipath signals, respectively. The direct signal will be a composite signal with a multipath signal and will be affected by these three multipath parameters. Figure 3 illustrates the signal correlation functions of an LOS multipath signal. Here, we can define the multipath amplitude ratio $\alpha_{\mathrm{LOS}}$ as follows:

(3) $$\alpha_{\mathrm{LOS}}=\frac{A_{1}}{A_{0}}$$

In Figure 3, the multipath amplitude ratio $\alpha_{\mathrm{LOS}}$ is 0.25. This multipath amplitude ratio $\alpha_{\mathrm{LOS}}$ is a major factor that distorts the correlation function of the LOS multipath signal. The smaller the amplitude of the reflection or diffraction signal with respect to the direct signal, the smaller the effect on the correlation function of the direct signal. In general, the first reflected signal has low power compared to a direct signal. The reflection and diffraction signals have a smaller amplitude $A_{1}$ than direct signals $A_{0}$ because they lose energy during reflection and diffraction. As a result, the shape of the correlated output of the LOS multipath signal with a direct signal becomes a clean triangular shape with only a single peak.

**Figure 4:**
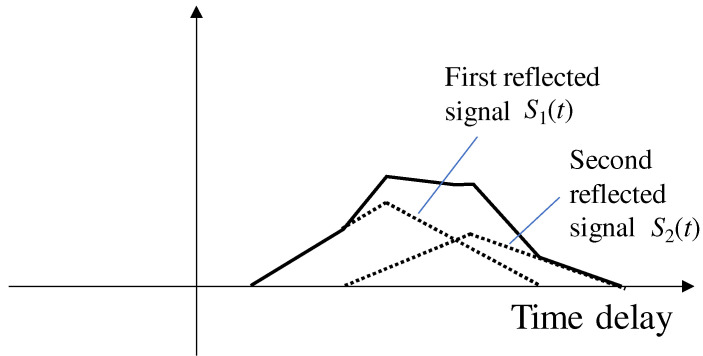
Correlation function of an NLOS multipath signal.

In the case of an NLOS correlation function, there is no direct signal, and the first reflected signal is distorted by the second reflected signal. Figure 4 illustrates the signal correlation functions of an NLOS multipath signal. The NLOS multipath signal can be expressed as follows:

(4) $$\begin{array}{cc} S_{\mathrm{NLOS}}\left(t\right) & =S_{1}\left(t\right)+S_{2}\left(t\right)\\ \; & =S_{1}\left(t\right)+A_{2}\cdot C\left(t-\tau_{2}\right)\cdot \cos\left(\omega_{0}t+\Delta \phi_{2}\right), \end{array}$$

The NLOS signal correlation function is a combination of the reflected or diffracted signal correlations. The multipath amplitude ratio of the NLOS signals $\alpha_{\mathrm{NLOS}}$ is defined as follows:

(5) $$\alpha_{\mathrm{NLOS}}=\frac{A_{2}}{A_{1}}$$

From Figure 4, the amplitude of the first reflected signal $A_{1}$ and second reflected signal $A_{2}$ are not considered to be significantly different. In the case of NLOS signals without a direct signal, the amplitude ratio $\alpha_{\mathrm{NLOS}}$ between the first and second signals is close to 1. Therefore, the multipath amplitude ratio between the LOS multipath signal $\alpha_{\mathrm{LOS}}$ and NLOS multipath signal $\alpha_{\mathrm{NLOS}}$ has the following relationship:

(6) $$\alpha_{\mathrm{LOS}}<\alpha_{\mathrm{NLOS}}$$

Therefore, the NLOS correlation function is more susceptible to the second signal than the LOS correlation function, resulting in a large distortion of the correlation function. For this reason, the NLOS correlation function does not have an ideal clean triangular shape.

We also consider the effect of the relative phase on the correlation function. From Equations (2) and (4), the shape of the signal correlation function depends on the phase of the second signal relative to the first signal. If the relative phase is 90${}^{\circ }$, the second signal does not affect the first signal’s correlation function. When the antenna is stationary, the phase of the reflected signal relative to the direct signal generally changes slowly as the satellite moves. The NLOS signal, by contrast, has a large relative phase variation owing to the complex synthesis of multiple reflections and diffraction signals. As a result, the correlation function of the NLOS signal is expected to become unstable over time.

We use these phenomena to detect NLOS signals. We created an NLOS classifier based on the machine learning of the features of the NLOS correlation shape. To realize machine learning from the features of the correlation shape, we extract the features of the NLOS correlation function using an actual dataset and use it to construct the NLOS classifier. However, a typical GNSS receiver only outputs a PVT solution. Therefore, we use a software GNSS receiver to obtain the signal correlation outputs in GNSS signal processing.

The next question is how to extract and discriminate the features of the NLOS from the distorted NLOS correlation output. In this study, we first extract the geometric features of the NLOS correlation output and attempt to classify it using an SVM. In addition, NLOS features are automatically extracted using an NN to identify the NLOS.

### 2.3. NLOS Detection Using SVM

As mentioned in the previous section, the direct and reflected signal correlation functions are combined in the LOS multipath signals. Consequently, the reflected signals distort the code correlation function of the direct signal, and the resulting code-tracking error causes an LOS multipath error. NLOS signal does not contain direct signals; the combined correlation function is more distorted than that in the case of LOS multipath signals.

We use multiple correlator outputs to extract NLOS features for SVM. GNSS incoming signal is multiplied by the in-phase (I) and quadrature (Q) locally generated carriers, and then the signals are correlated with a slightly shifted PRN code generated in the GNSS receiver. We use the multi-correlator structures; thus, 2J+1 correlation points are computed. We use the in-phase component of correlation outputs for SVM. We consider the perfect Doppler frequency compensation, and the *j*th in-phase correlator output at time *k* is represented as follows:

(7) $$I_{j,k}=A_{k}R\left(\tau_{e,k}+\delta_{j}\right)\cos\left(\phi_{e,k}\right)$$

where j=−J,…,J, $\delta_{j}$ is the correlator delay. R(·) is the auto-correlation function. $\tau_{e,k}$ and $\phi_{e,k}$ is the error of the code delay and the carrier phase estimated at the receiver. The set of multi-correlator outputs per 1 ms at time *k* is as follows:

(8) $$C_{k}=\left\{I_{-J,k},\ldots ,I_{0,k},\ldots ,I_{J,k}\right\}$$

In developing a successful SVM classifier, appropriate feature extraction is very important. We extract the features of the NLOS signals from multi-correlator outputs for SVM to construct the NLOS classifier. In this study, we extract the following features:

- Signal strength versus elevation angle
- Number of local maxima of the correlation outputs
- Distribution of the delay of the maximum correlation

**Figure 5:**
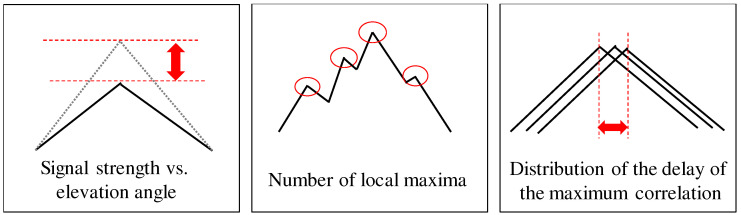
Three types of NLOS features are extracted from GNSS signal correlation outputs and are used for NLOS classification applying a support vector machine (SVM).

Figure 5 illustrates an overview of these three NLOS features to classify the NLOS signals from the GNSS correlation outputs. We describe the details of these features in the following section.

#### 2.3.1. Signal Strength versus Elevation Angle

The magnitude of the correlation peak is related to the GNSS signal strength. In general, the signal strength of the reflected or diffracted signals is weaker than that of the directly received signals. As a result, the signal strength can be used to detect NLOS signals. Even in ordinary GNSS positioning calculations, the SNR, which represents the GNSS signal strength, is widely used to exclude NLOS multipaths and select satellites for the positioning calculations [7,8]. Because the signal strength of GNSS is considered to be a good representation of the NLOS signal, the maximum value of the correlation ground is adopted as the NLOS feature in this study. However, the signal strength is also dependent on the satellite elevation angle. We model the maximum correlation output in an open-sky environment using the actual observed data to evaluate the NLOS possibility. The polynomial function that represents the relationship between the satellite elevation, and the maximum correlation value is determined using a regression from the actual data obtained in an open-sky environment. Here, we express the open-sky maximum correlation value $\eta (\theta_{el})$ as a fourth-order polynomial at an elevation angle with an approximate maximum correlation value as the coefficient.

(9) $$\eta \left(\theta_{el}\right)=a_{4}\theta_{el}^{4}+a_{3}\theta_{el}^{3}+a_{2}\theta_{el}^{2}+a_{1}\theta_{el}+a_{0}$$

The coefficients $a_{0}$ to $a_{4}$ are then estimated using the least-squares method.

The NLOS feature $F_{1,t}$ based on the signal strength versus the elevation angle at time *t* is calculated as follows:

(10) $$F_{1,t}=\frac{1}{M}\sum_{k=t-M+1}^{t}\left\{\frac{\max\left(∥C_{k}∥\right)}{\eta (\theta_{el,k})}\right\},$$

where *M* is the total number of correlations that are non-coherently integrated. The value of $F_{1,t}$ is expected to be small when the observed signal is an NLOS signal. We use this NLOS feature for machine learning.

#### 2.3.2. Number of Local Maxima of Correlation Outputs

**Figure 6:**
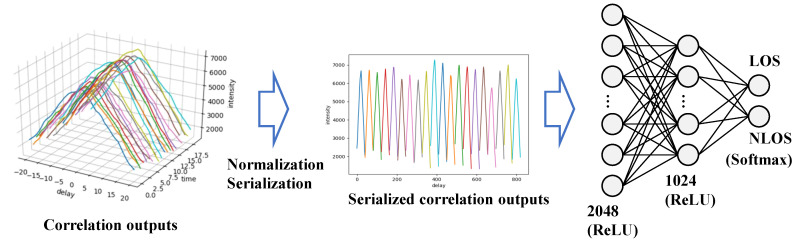
Overview of the proposed NLOS detection system using a neural network (NN). Serialized correlation outputs are input to a fully connected layer of the NN. Here, ReLU is an activation function, and the softmax is a function that outputs the probability. The probability of the LOS and NLOS signals is output of the NN.

According to Figure 6, there are multiple peaks in the NLOS correlation function. In the case of an NLOS signal, the correlation function comprises a combination of the correlation functions of only the reflection and diffraction signals. The correlation function of the first reflection/diffraction signal is distorted by that of the second reflection/diffraction signal. As a result, the symmetry correlation shape collapses, and multiple local maxima of the correlation outputs appear. It is expected that the number of local maxima of the NLOS correlation outputs is greater than that of the LOS correlation outputs. The number of local maxima of the correlation outputs was directly used for the classification. The NLOS feature $F_{2,t}$ is calculated as follows:

(11) $$F_{2,t}=\frac{1}{M}\sum_{k=t-M+1}^{t}\left\{local\_maximum\left(∥C_{k}∥\right)\right\}$$

where local_maximum(·) is a function that counts the number of local maxima. As the NLOS feature, we use the average number of local maxima *M* times. It is expected that the number of local maxima is almost 1 in the case of LOS signals. By contrast, the number of local maxima will increase in the case of NLOS signals.

#### 2.3.3. Distribution of Delay of Maximum Correlation Outputs

In the LOS correlation function from Figure 6, the delay of the maximum correlation output is almost zero. However, in the case of the NLOS signal, the delay of the maximum correlation output has a large distribution. We compute the variance of the delay used as the NLOS feature. The NLOS feature $F_{3,t}$ is calculated as follows:

(12) $$F_{3,t}=\frac{1}{M}\sum_{k=t-M+1}^{t}\left({\hat{\tau }}_{e,k}-\bar{\tau }\right)$$

where ${\hat{\tau }}_{e,k}$ is the estimated delay of the maximum correlation output and $\bar{\tau }$ is the mean of the estimation delay *M* times. It can be concluded that the variance of delay of the NLOS correlation output is greater than that of the LOS signal.

#### 2.3.4. SVM

In constructing the NLOS signal classifier, we use an SVM, one of the supervised machine learning algorithms [32]. SVM uses a kernel trick technique to transform the input data to find the optimal bounds of the target classes to classify nonlinear data. Although various SVM kernels have been proposed, we use the radial basis function kernel (RBF), which is the most general and versatile. We chose RBF kernel and a linear decision function because the extracted NLOS features do not always allow linear separation. There are two parameters associated with RBF kernel, cost and kernel parameter. These parameters are selected by a grid search approach [33]. We compare the discriminator trained using the propsosed NLOS features and an SVM with the discriminator using the neural networks described in the next section.

### 2.4. NLOS Detecition Using NN

We employ neural networks to construct NLOS signal classifiers. A neural network is a computational model inspired by the manner in which biological neural networks in the human brain process information. A signal *y* output from a neuron is represented by an input element $x_{i}$ and a corresponding weight $w_{i}$ and bias *b*.

(13) $$y=f\left(\sum_{i=1}^{N}x_{i}w_{i}+b\right)$$

The neuron fires when the sum of the weighted inputs exceeds a threshold. A function that determines how to fire an input signal is called an activation function f(·), by which the input for the next neuron is determined. A neural network consists of a combination of multiple neuron models. The weights $w_{i}$ and bias *b* are adjusted using the backpropagation method in the neural networks. For the backpropagation method, a loss function for calculating the difference between the output and correct value is defined, and the gradient is optimized to be minimized between the output and input layers.

A neural network can be decomposed into an input layer, a hidden layer, and an output layer. In the classification case, the number of output layer units is the number of classification classes. Because this research classifies LOS and NLOS signals, there are two output layers. Figure 6 illustrates the outline of the proposed NLOS signal classifier neural network model.

First, as a preprocessing of the input data, we normalize the output of the GNSS signal correlation values using the relationship between the satellite elevation angle and the signal strength. To remove the variation in amplitude owing to the satellite elevation angle, similar to with an SVM, Equation (9) is used. The M·(2J+1) correlated outputs are aligned and serialized to input into the NN. We used a simple neural network with two hidden layers. We use the ReLU function as the activation function in the hidden layer. Furthermore, we use the softmax function in the output layer. The softmax function is a function that outputs the probability with the class total being 100%. This is employed when outputting a probability. We train this NN model using cross-entropy as the loss function. We can expect to automatically learn NLOS features from GNSS correlation waveforms through the training of this NN. However, learning with an NN requires a large number of supervised data. In this study, the correlation of the value outputs labeled as LOS and NLOS is required. In the next section, we describe how to efficiently collect the training data.

## 3. Collection of Training Data

In this study, we create an NLOS detection classifier using an SVM and an NN. We use the shape of the GNSS correlation outputs to detect the NLOS signals. There are many types of signal correlators, such as early late correlators, narrow correlators, and double delta correlators. The correlation shape, which is the output of the correlators, depends on the number of correlation points and the sampling frequency of the front-end. The receiver bandwidth also affects the correlation shape. In the consumer’s GNSS receiver, these correlator models are a total black box, and we cannot obtain the correlation output. Thus, we use a software GNSS receiver to address this problem. Software receivers are widely recognized and used in GNSS research because of their configurational flexibility and ease of use. We use a self-designed GNSS signal correlator by applying a software GNSS receiver and use a front-end (NSL STEREO, UK) GNSS-SDRLIB, which is an open-source software GNSS receiver [34]. The sample rate adopted in this test was 20 MHz. We computed the GNSS LOS and NLOS correlation outputs using 21 correlation points.

To obtain the LOS and NLOS reference data, we use fish-eye images and verify the NLOS signals from the images. A large number of training data are required for machine learning, which are not easy to collect through a manual check of the fish-eye images. Furthermore, to obtain accurate LOS and NLOS references, accurate azimuth angle information of a fish-eye camera is required to project the satellite position onto the fish-eye image. Therefore, in this study, we created a device that automatically collects the NLOS reference signals for training.

**Figure 7:**
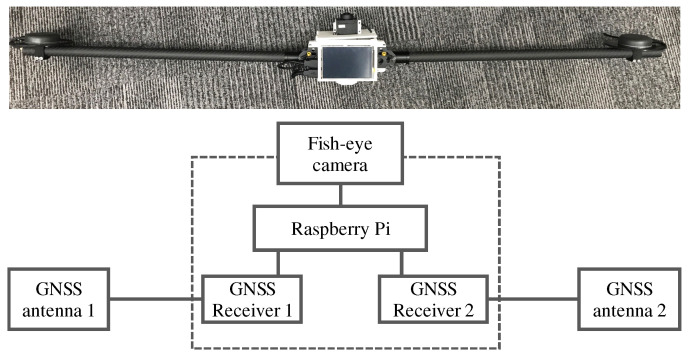
We developed an automatic NLOS training data acquisition device. We used a fish-eye camera and two GNSS antennas/receivers to determine the NLOS satellite for SVM and NN training.

**Figure 8:**
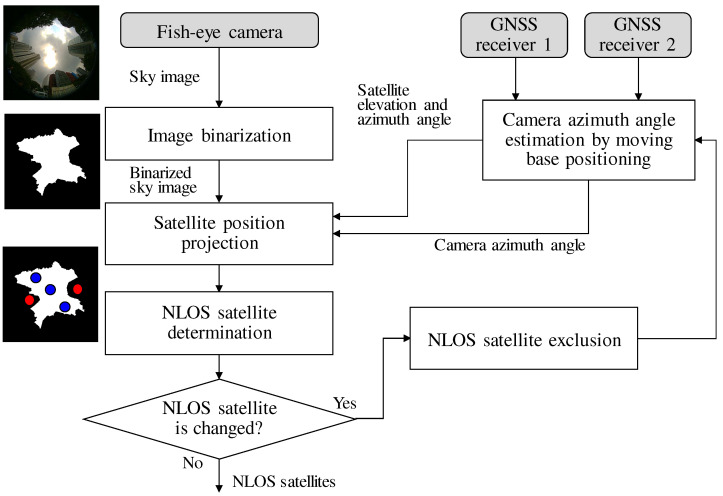
Flow of NLOS satellite determination using a fish-eye image. We iteratively calculate the azimuth angle of the camera and determine the NLOS satellite from the fish-eye image.

Figure 7 shows the device used to automatically determine and collect the NLOS signals. The device consists of a fish-eye camera, two pairs of GNSS antennas, and a receiver. To project the satellite position on the image taken by a fish-eye camera, it is necessary to measure the azimuth angle of the camera at the time the image is taken. Therefore, we use moving-base positioning between two GNSS pairs to estimate the azimuth angle of the camera. Moving-base positioning is one of the GNSS relative positioning methods used to estimate the relative vectors between antennas by calculating the difference between the carrier phase of two sets of GNSS observations, as well as the normal RTK-GNSS [35]. However, in an environment where NLOS multipathing occurs, the effect of multipathing causes errors in the moving-base positioning. We solve this problem by using an iterative NLOS exclusion method. The flow used to determine the NLOS signals for SVM and NN training is presented in Figure 8.

The flow of the NLOS detection using a fish-eye image is as follows:

(i) First, the device captures a sky image using a fish-eye camera. Two GNSS receivers record GNSS raw observations (pseudoranges, carrier phases, and navigation messages).
(ii) Second, the sky image is binarized to recognize buildings that constitute obstacles to a direct signal. A binarized sky image can be used as a mask to determine the NLOS signal.
(iii) Next, the elevation and azimuth angle of the satellites are computed through GNSS positioning. The azimuth angle of the fish-eye camera can then be computed by the moving-base positioning using two GNSS antennas/receivers. Note that the estimated azimuth angle may contain errors owing to the effect of the NLOS multipath signals.
(iv) We project the satellite position onto a binarized fish-eye image using the estimated azimuth angle of the fish-eye camera.
(v) The satellite visibility is automatically determined based on whether the buildings and satellite are overlapped. The temporal NLOS satellites can be determined.
(vi) We then exclude the NLOS satellites and again estimate the azimuth angle of the fish-eye camera using the moving-base positioning. By excluding the NLOS satellite, the correct camera azimuth can be expected to be estimated.
(vii) We repeat the process of (iii) through (vi) until the estimated azimuth angle of the fish-eye camera converges.

Finally, we can obtain a reference for the correct satellite visibility. We collected the actual LOS and NLOS GNSS correlation outputs using this device to create an NLOS signal classifier.

## 4. Experiments

### 4.1. Experimental Environment and Setting

**Figure 9:**
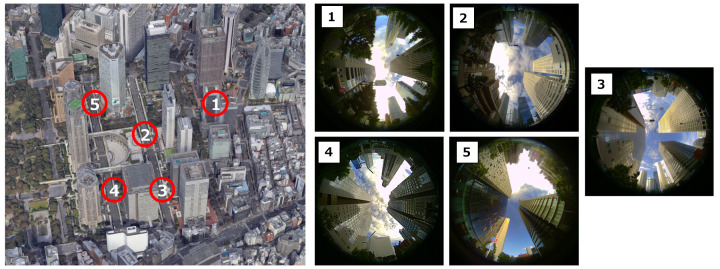
Experimental environment and sky images. We collected GNSS signal correlation outputs in five different locations in the Shinjuku.

We evaluated the NLOS classification performance of the proposed method. The experiments were conducted in actual urban environments (Shinjuku area, Tokyo, Japan). Figure 9 shows the experimental environment and the captured sky image at each location. In this environment, as shown in Figure 9, there are many high-rise buildings of over 100 m, and NLOS multipath signals frequently occur. The training data were collected at five locations in Figure 9 using the GNSS software receiver. We used the NLOS training data acquisition devices to acquire three sets of 5 min signal correlation data every two hours at each location. Because the location of the satellites varies significantly over time, we obtained a variety of GNSS LOS and NLOS signal correlation outputs and used them for training. The acquired signal correlation outputs are labeled LOS/NLOS, and we trained the discriminator using the proposed SVM and NN models. We evaluated the classification performance using the cross-validation method. We repeat the process using the data set in four of five different locations for training and the remaining one data for an evaluation of the mean of the classification rate.

We employed MATLAB to construct the NLOS signal classifier using an SVM and an NN. The parameters set for machine learning are listed in Table 1. The normalized correlation output was used as input to the SVM and NN, and 20 ms correlation outputs were used for training. Determining the hyperparameters is a challenging problem. In the case of SVM, the cost and kernel parameters were selected by a grid search approach [33]. In the case of NN, the hyperparameters, such as network structure and learning rate were determined empirically through trial and error. As for the layer structure and learning rate of the NN, we tested combinations of one, two, and three fully connected layers and learning rates of 0.1, 0.01, and 0.001. Among these combinations, we adopted two fully connected layers and 0.01 learning rate, which had the best classification performance.

### 4.2. Correlation Outputs

**Figure 10:**
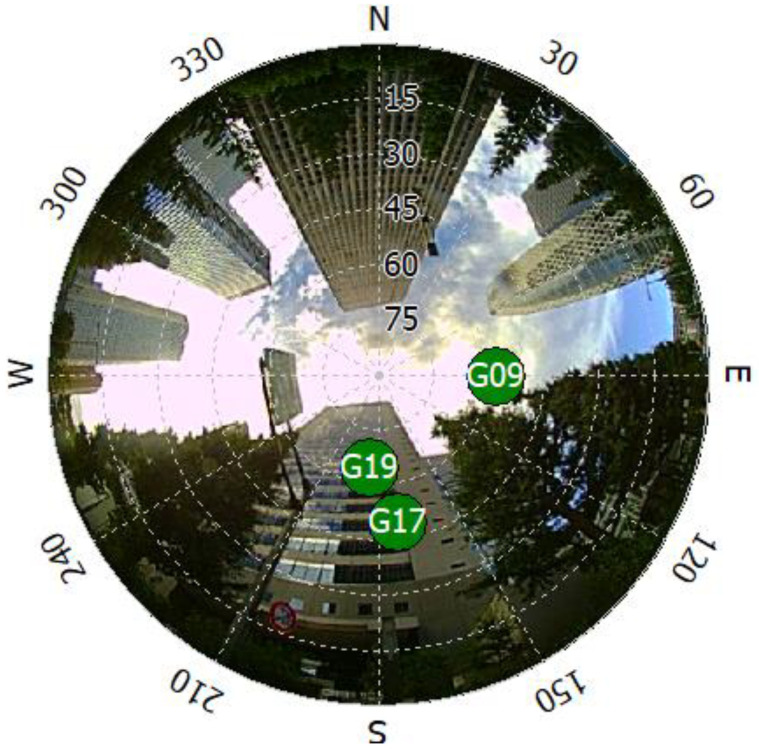
GPS satellite projection on a fish-eye image.

**Figure 11:**
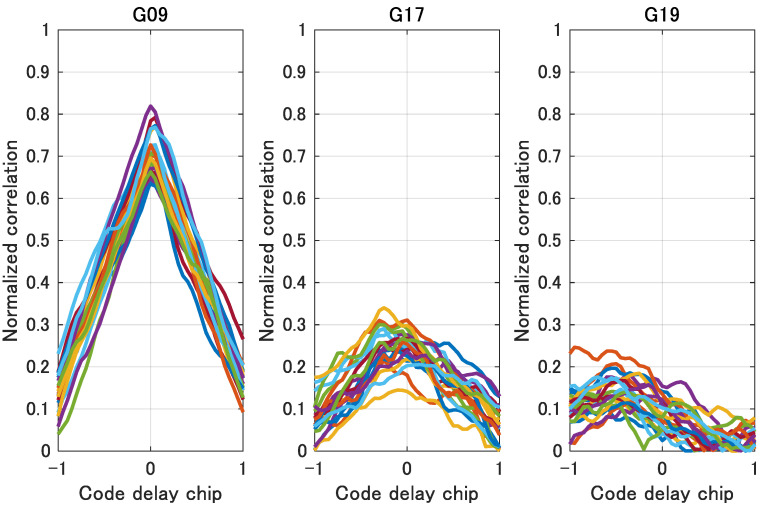
Examples of LOS and NLOS signal correlation outputs. 1 ms correlation outputs for a 20 ms period are overlapped in different colors. The left figure SHOWS the correlation outputs of LOS satellites, and the two figures on the right show the correlation outputs of NLOS satellites.

The actual correlation output was obtained and visualized to investigate the extraction of NLOS features from the correlation output of NLOS signals. Figure 10 shows a fish-eye image of the Test #1 environment in Figure 11 and the actually received GPS satellite position projected on the fish-eye image. Figure 10 shows that GPS satellite “G09” is LOS and “G17” and “G19” are NLOSs.

We computed the GPS LOS and NLOS correlation outputs. Here, the sampling frequency and bandwidth of the RF front end are 20 and 4.2 MHz, respectively. Figure 11 shows the 1 ms correlation outputs of the LOS and NLOS signals overlapping for 20 ms. Figure 11 shows that the NLOS signal correlation functions (“G17” and “G19”) do not have a clear peak at the center, and a plurality of local maxima exists at a point other than the peak. Furthermore, the NLOS signals have a smaller correlation peak than the LOS signals even for a satellite with the same elevation angle. Figure 11 shows that the shape of the correlation function of the actual NLOS signal is geometrically different from the shape of the LOS correlation function.

**Figure 12:**
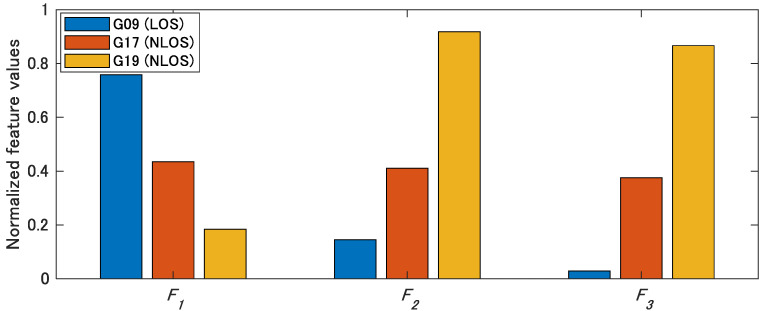
Evaluation of the extracted NLOS features of SVM. NLOS features $F_{1}$, $F_{2}$, and $F_{3}$ are calculated from Equations (10)–(12), respectively. There is a difference between the LOS and NLOS satellites in all features.

We compute the proposed NLOS features for SVM from the actual correlation outputs. Figure 12 shows the normalized NLOS features $F_{1}$, $F_{2}$, and $F_{3}$ computed from Equations (11)–(13), respectively. NLOS feature $F_{1}$, which is based on the signal strength, has a smaller value when the satellite is NLOS signals. NLOS features $F_{2}$ and $F_{3}$ of the actual NLOS satellites “G17” and “G19” are larger than the features of the LOS satellites “G09”. SVM is a classification method based on input features. In all features, there is a difference between the LOS and NLOS satellites. This difference indicates that LOS and NLOS can be separated by SVM, and the three features correctly contribute to the classification by SVM.

**Figure 13:**
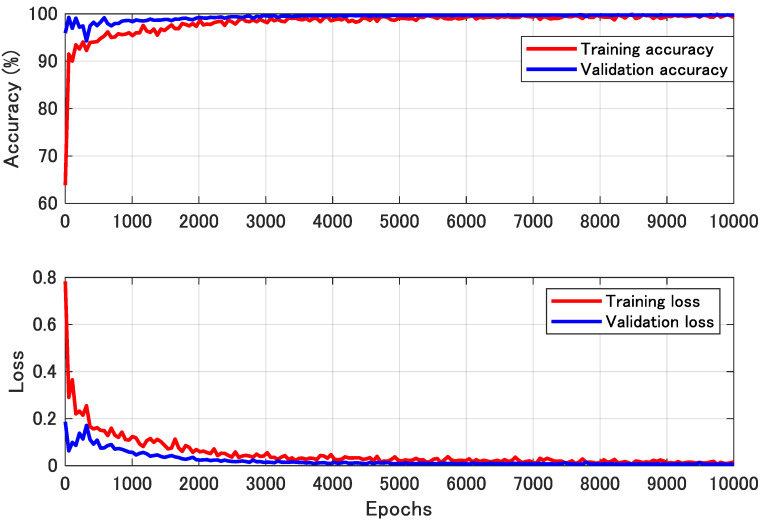
Learning curve of NN. The upper figure shows the training and validation accuracy and lower figure shows the loss.

Figure 13 shows an example of the learning curve of the NN training process. The upper figure in Figure 13 shows the training and validation accuracy, and the lower figure shows the training and validation loss. The accuracy of the training data is converged to 100%, and the accuracy of the validation data is also converged to 100% without any decrease. Furthermore, there is no significant gap between training and validation, which indicates that learning is proceeding without overfitting.

### 4.3. Classification Results

We compared a general satellite selection method using the GNSS SNR threshold with the two proposed methods. The SNRs of the reflected and diffracted NLOS signals are typically lower than those of the direct signals. The SNR threshold was set to −5 dB from the SNR–elevation curve shown in Equation (9). When the SNR of the input signal is lower than the individual threshold, it is judged as an NLOS signal.

**Figure 14:**
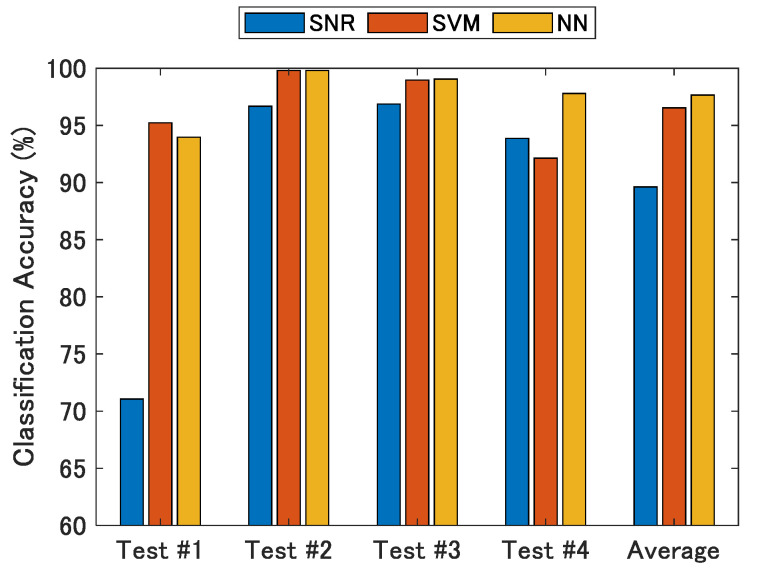
Classification accuracy using SNR, SVM, and NN. NN has the best performance among the three proposed classifiers.

Figure 14 shows the results of the NLOS classification accuracy when using the proposed methods. The NLOS classification based on the SNR has the lowest accuracy, whereas the SVM and NN have almost the same classification accuracy. It can be confirmed that the proposed NN has the best classification performance from the average classification accuracy of all tests in Figure 14. Comparing the SVM with the NN, the classification accuracy of the NN was slightly higher, and the NN had the highest classification accuracy for all experimental data except for test #1. The average classification rate at which a signal from the NLOS signal group was correctly classified as the NLOS signal was 97.8% when using the proposed NN.

We used typical evaluation statistics used in binary classification. We calculated true positive (*TP*, correct detection), true negative (*TN,* correct rejection), false negative (*FN*, an omission error), and false positive (*FP*, a commfission error) in each classification test. We then calculated the average classification rate (*A*), average recall (*R*), average precision (*P*), and average *F*-Measure (*F*) using the following equations [36].

(14) $$A=\frac{TP+TN}{TP+TN+FP+FN}$$

(15) $$R=\frac{TP}{TP+FN}$$

(16) $$P=\frac{TP}{TP+FP}$$

(17) $$F=2\frac{R\cdot P}{R+P}$$

**Figure 15:**
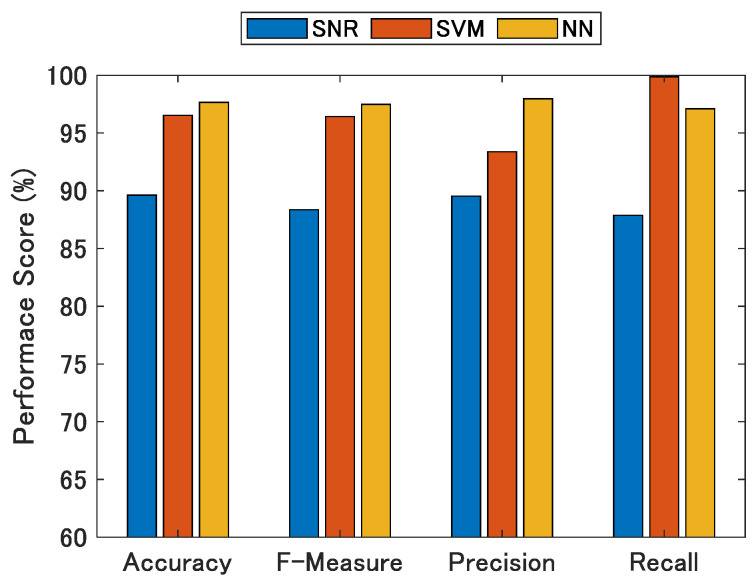
Performance of NLOS classifiers using SNR, SVM, and NN.

Figure 15 show the evaluation statistics. Here, the proposed NN classifier achieves good results for all performance measures in comparison to all other classifiers. From the viewpoint of the positioning accuracy, it is important for the recall of the NLOS signal discrimination to be close to 100% because the positioning accuracy significantly deteriorates when the NLOS signal is used for positioning. These results show that the recall was close to 100% for both the SVM and NN, and that most of the NLOS signals can be detected.

## 5. Discussion

The NLOS detection methods using machine learning for the GNSS signal correlation values could discriminate the NLOS with much higher accuracy than a method using the GNSS SNR threshold. We believe that this was because we had extracted the appropriate features of the NLOS correlation outputs by SVM and NN. In the cross-validation results from Figure 14, the classification accuracy of Test #1 using the SNR threshold was worse than the that of the other tests. The training model is dependent on the training location because we used the data set in four of five different locations for training in the cross-validation test. There is a possibility that Test #1 includes the NLOS signals with high SNR compared with other locations. As a result, the traditional method based only on SNR could not correctly classify the NLOS signal. In contrast, SVM and NN show high classification accuracy even in Test #1. The cross-validation results also show that even when Test #1 was included in the training (Test #2, #3, and #4), LOS and NLOS could be identified without any problem. This suggests that the proposed method has sufficient generalization performance.

Comparing the results of NN and SVM, the performance of NN was slightly higher. This result suggests that the NLOS features extracted by NN using the signal correlation values directly were more effective than the NLOS features designed by humans.

The data presented in this paper were obtained in the same city, and it has not been verified whether the same model can be applied to different cities. In the future, we plan to check whether the model learned across different cities has sufficient generalizability. If the model is to be applied to cities with completely different shapes and distributions of buildings, it is thought that it may be necessary to add training data obtained in those cities.

In terms of the computational cost, an NN has a higher computational cost than an SVM. During the training stage of the NNs, the computation time depends on various hyperparameters, although it is 5 to 10 times longer than training with SVM. During the test stage, the average processing time required for 100 samples in the current model using MATLAB was 0.66 ms for the SVM and 1.27 ms for the NN. Both methods can be executed in real-time; however, because an SVM is less computationally expensive, in cases in which the computational resources are limited, an SVM is considered more effective than an NN.

## 6. Conclusions

We proposed NLOS multipath detection methods that can employ machine learning techniques and software-based GNSS receiver to enhance the GNSS positioning performance in urban environments, where NLOS multipath signals lead to major positioning errors. We employed SVM and NN to construct the NLOS signal classifier in this study. In the SVM, we extracted the three features of the shape of the multi-correlator output. In the NN, we directly input the multi-correlator output to construct the NLOS classifier. The NLOS classification experiments in actual urban environments show that the NN has slightly better classification performance than SVM, and 97.7% of the NLOS multipath signals were correctly discriminated. In future studies, we plan to collect more training data for the NLOS classifier to improve the NLOS classification ratio and create classifiers for satellites other than those used by GPS.

In this study, we evaluated the discrimination performance using a cross-validation method. However, the data are all taken within the same city (Tokyo). To further evaluate the generality of the proposed method, it is necessary to evaluate the NLOS discrimination method using data obtained from different cities and to evaluate the NLOS discrimination of mobile vehicles in the future.

## Figures and Tables

**Table 1:** Parameters for SVM and NN. The Cost parameter, Kernel parameter, and learning rate are the hyperparameters of SVM and NN.

Model	SVM	NN
Input	Normalized correlation outputs Size: 21 × 20	Normalized correlation outputs Size: 21 × 20
Output	2 classes (LOS/NLOS)	2 classes (LOS/NLOS) with probability
Cost parameter	1	-
Kernel parameter	0.01	-
Batch size	-	1024
Learning rate	-	0.01

## Abbreviations

The following abbreviations are used in this manuscript:

GPS	global positioning system
GNSS	global navigation satellite system
LOS	line-of-sight
NLOS	non-line-of-sight
SNR	signal-to-noise ratio
SVM	support vector machine
NN	neural network
PVT	position, velocity, and time
PRN	pseudo-random noise
